# Safe nasendoscopy assisted procedure in the post‐COVID‐19 pandemic era

**DOI:** 10.1111/coa.13591

**Published:** 2020-06-25

**Authors:** Ajith George, Mark Prince, Chris Coulson

**Affiliations:** ^1^ University Hospital North Midlands Stoke on Trent UK; ^2^ Endoscope‐i Ltd Birmingham UK; ^3^ University Hospitals Birmingham Birmingham UK

**Keypoints**

- Droplets produced by coughing or sneezing carry a higher viral particle load and can be reduced by wearing a surgical mask in turn helping reducing infection transmission.
- The SNAP device is an effective and safe method of providing access to the nasal cavity during nasoendoscopy whilst also providing a barrier of a surgical mask to protect against droplet dispersion.
- Reducing the exposure to pathogens for all healthcare works facilitates a return of diagnostic procedures for the nose and throat after the COVID‐19 pandemic peak.

For centuries, it has been humankind's instinct to cover the mouth and nose when coughing or sneezing. Common sense would dictate this instinctively reduces the dispersion of aerosol and droplets and thus the spread of contact and airborne infections.

Aerosol generating procedures (AGPs) have become a new byword for procedures that put clinicians at increased risk of contracting COVID‐19. Whilst the title suggests the risk is simply in aerosols, the science is much more interesting. Droplets and aerosols are different, with the distinction between them based on size. Whilst experts disagree on the absolute size when an aerosol becomes a droplet, the general acceptance is that anything bigger than 50 microns (0.05 mm) is a droplet and those smaller are aerosols.

In most contagious respiratory infections, the principal transmission agents are droplets.^1^ This is due to the relatively high viral load in a droplet, purely due to its large size, and also the fact that large droplets have weight, and so gravity pulls them down onto surfaces that others can touch—so passing it on. This is why washing hands is so effective against droplet spread.

Aerosol transmission is thought to be a much less frequent cause of transmission, mainly due to the very small viral load (given the aerosol itself is by definition very small). However, it is clearly more concerning as these very light particles can travel large distances. That said, it is thought to only play a minor role in transmission compared to droplet spread.

During the COVID‐19 pandemic, PHE (Public Health England) updated guidance on what it considers (AGPs) Aerosol Generating Procedures. Included within this list were examinations of the upper aerodigestive tract in ENT. Healthcare workers were recommended to reduce endoscopy of the nose and throat and essential examination had to be performed using high level PPE including an respirator (N95 or FFP3).^2^

Anfinrud et al^3^ graphically represented a visual reduction in aerosol production by creating light sheet from a 532‐nm green LASER. Comparisons were made between a person talking with and without a cover for the mouth, in their instance, a slightly dampened washcloth. Light flashes were recorded to evaluate the number of droplets ranging between 5‐200 microns. They showed that by covering the mouth, virtually no light flashes were seen. This observation supports the well‐known concept that covering the mouth does indeed reduce droplet production.

On impact with smooth surfaces, droplets disperse to smaller sizes and can aerosolise. Similarly, impact onto soft surfaces absorbs droplets reducing their projection as well as the tendency to aerosolise.^3^

As the pandemic plateaus in countries across the world, various strategies are to be considered to return to a new normal. This would facilitate the resumption of diagnostic services whilst maintaining the protection to healthcare workers. One suggestion is the use of facemasks to help reduce the risk of inadvertent droplet dispersion.^2^ Despite the “soft surface” barrier masks create, in the ENT setting, facemasks obscure access to the nasal cavity thus preventing nasoendoscopy.

The “SNAP” (Safe Nasoendoscopic Airway Procedure) developed by endoscope‐i Ltd (West Midlands, UK) is a single‐use, valved endoscopic port, retrofitted to any surgical mask (Figure 1), permitting entry of a 4 mm flexible and rigid endoscope to examine the naso and pharyngolarynx. The valve, a 10.9‐mm cylindrical tube truncated by two opposing 45 degree inclined membranes 700 microns thick, approaches a point but terminates in a 700 micron thick and 500 micron wide plateau, creating a “duck bill” formation. The valves are formed using a FFF (fused filament fabrication) 3D printing technique with a Flashforge Creator Pro 3D printer. The plateau atop the valve serves to provide a reference for introducing a slit using a hardened steel razor blade that is 400 microns thin. The blade is mounted in a jig to ensure angle, penetration depth and position are controlled as it is driven through the membrane. These measures ensure that the valve opening is observably consistent and less than 50 microns.

**Figure 1:**
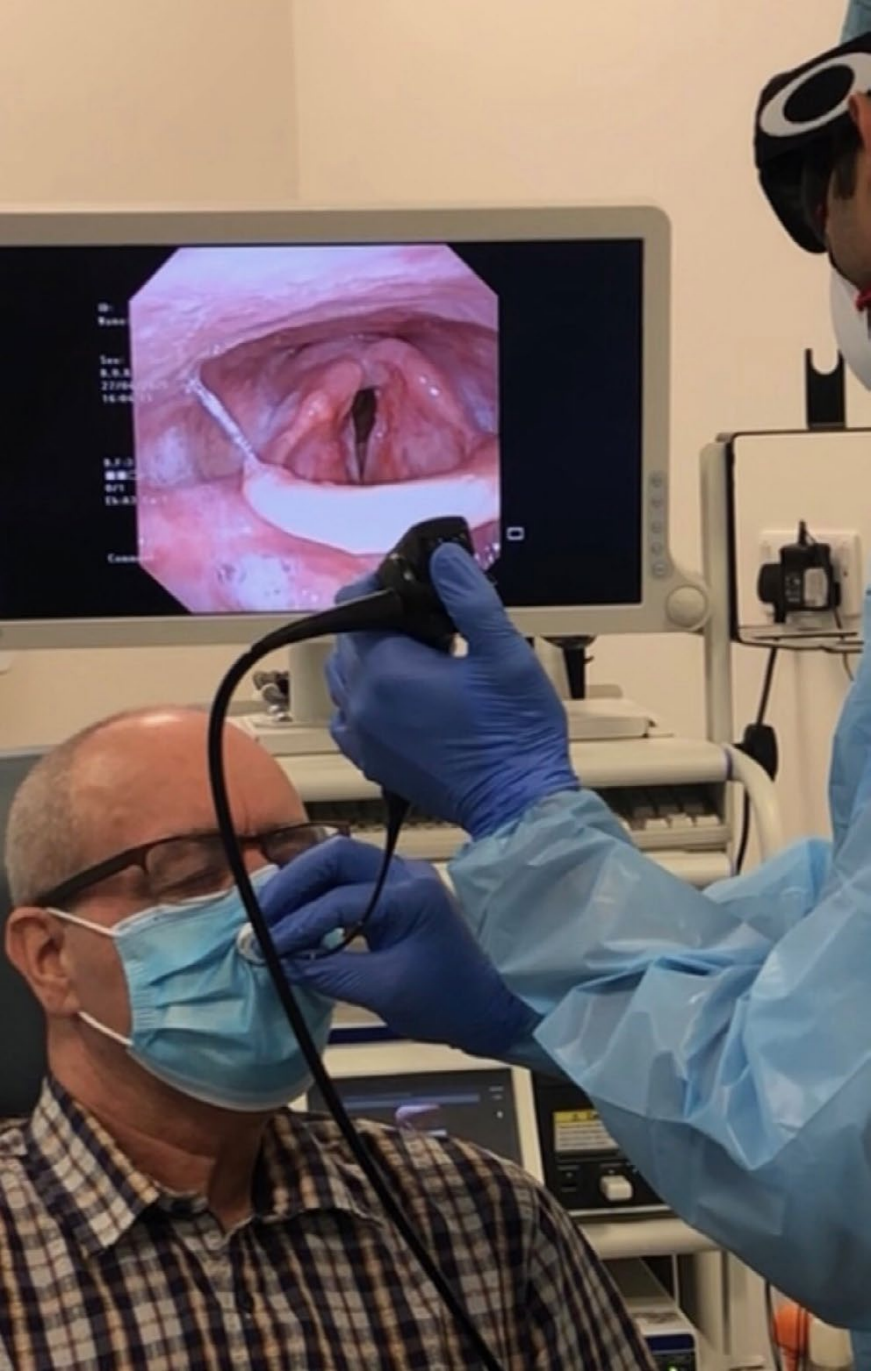
The SNAP mask in use on a patient during flexible laryngoscopy (permission granted for publication)

Once the SNAP is fitted to a surgical mask, any cough or sneeze generated during the procedure is caught within the mask. The valve is configured such that pressure from the patient side will serve to collapse the walls of the valve membrane thereby further sealing the slit in the valve. This seal has been in vitro tested with aerosolised fluorescein (Figure 2) on a Kimberly Clark Tecnol Procedure Mask (Halyard, UK). The 45‐degree angle of the valve walls from the non‐patient side similarly allows the blunt tip of the nasoendoscope to deform the valve walls with ease. The cylindrical form of the walls encourages the valve membranes to return to their original flat shape following withdrawal of the endoscope.

**Figure 2:**
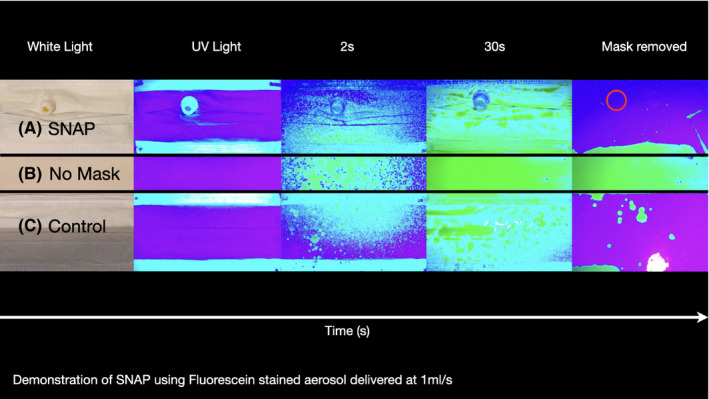
In Vitro Aerosolised Fluorescein test results 1ml/s through a 50 micro point. From superior to inferior: (A) “mask fitted with SNAP” vs. (B) “no mask” vs. (C) “mask with no SNAP” as positive control to demonstrate no leakage through the mask material. No visual difference seen and no leak through the SNAP valve (red circle)

During the COVID‐19 pandemic, our tertiary head and neck cancer referral centre managed 120 urgent 2ww cases. Using the Tikka et al calculator,^4^ 40% of referrals were redirected back to the GP. The remaining 60% either went direct for imaging or underwent endoscopy. In total, 40 cases were endoscoped, nine of which using the SNAP. All 9 cases scoped with the SNAP were completed without any adverse effect. No cough or sneeze was elicited during any of the examinations, and observations between the two groups were identical. Subsequently, one consultant lead FEES examination was performed under controlled conditions. Again, the procedure was completed without any complications. The patient self‐remarked on the comfort of the endoscopy as a result of the stability provided by the SNAP device in the alar region which prevented inadvertent movement during the chin‐tuck and head‐turn manoeuvers.

Our observations demonstrate the SNAP device is a practical and safe tool to aid reduction in droplet dispersion whilst performing nasoendoscopy. We hope to see the inclusion of such a device in recovery guidelines by national bodies in order to facilitate the return of safe nasoendoscopy in the post‐COVID pandemic era.

## CONFLICT OF INTEREST

All three authors have declared and signed the COI form stating their involvement in endoscope‐I Ltd who have patented, designed and created the SNAP.
